# West Nile virus emergence in humans in Extremadura, Spain 2020

**DOI:** 10.3389/fcimb.2023.1155867

**Published:** 2023-07-04

**Authors:** Alicia Macias, Paloma Martín, Mayte Pérez-Olmeda, Beatriz Fernández-Martínez, Diana Gómez-Barroso, Esperanza Fernández, Julian Mauro Ramos, Laura Herrero, Saray Rodríguez, Elena Delgado, Maria Paz Sánchez-Seco, Miguel Galán, Antonio Jesús Corbacho, Manuel Jimenez, Cristian Montero-Peña, Antonio Valle, Ana Vázquez

**Affiliations:** ^1^ Servicios de Microbiología y Medicina Interna, Hospital Don Benito-Villanueva de la Serena, Don Benito, Badajoz, Spain; ^2^ Servicio de Microbiología , Hospital Universitario de Badajoz, Badajoz, Spain; ^3^ Centro Nacional de Microbiología, Instituto de Salud Carlos III (CNM-ISCIII), Madrid, Spain; ^4^ CIBER de Enfermedades Infecciosas (CIBERINFEC), Madrid, Spain; ^5^ Centro Nacional Epidemiología, Instituto de Salud Carlos III (CNE-ISCIII), Madrid, Spain; ^6^ CIBER de Epidemiología y Salud Pública (CIBERESP), Madrid, Spain; ^7^ Banco de Sangre de Extremadura, Junta de Extremadura, Mérida, Badajoz, Spain; ^8^ Subdirección de Epidemiología, Servicio Extremeño de Salud, Mérida, Badajoz, Spain; ^9^ Servicio Medicina Familiar y Comunitaria, Centro de Salud Don Benito Oeste, Hospital Don Benito-Villanueva, Don Benito, Badajoz, Spain

**Keywords:** West Nile virus, human infection, flaviviruses, diagnosis, molecular and serological methods, surveillance

## Abstract

In Spain, the largest human West Nile virus (WNV) outbreak among humans was reported in 2020, constituting the second most important outbreak in Europe that season. Extremadura (southwestern Spain) was one of the affected areas, reporting six human cases. The first autochthonous human case in Spain was reported in Extremadura in 2004, and no other human cases were reported until 2020. In this work, we describe the first WNV human outbreak registered in Extremadura, focusing on the most important clinical aspects, diagnostic results, and control actions which followed. In 2020, from September to October, human WNV infections were diagnosed using a combination of molecular and serological methods (an in-house specific qRT-PCR and a commercial ELISA for anti-WNV IgM and IgG antibodies) and by analysing serum, urine, and/or cerebrospinal fluid samples. Serological positive serum samples were further tested using commercial kits against related flaviviruses Usutu and Tick-borne encephalitis in order to analyse serological reactivity and to confirm the results by neutralisation assays. In total, six cases of WNV infection (five with neuroinvasive disease and one with fever) were identified. Clinical presentation and laboratory findings are described. No viral RNA was detected in any of the analysed samples, but serological cross-reactivity was detected against the other tested flaviviruses. Molecular and serological methods for WNV detection in various samples as well as differential diagnosis are recommended. The largest number of human cases of WNV infection ever registered in Extremadura, Spain, occurred in 2020 in areas where circulation of WNV and other flaviviruses has been previously reported in humans and animals. Therefore, it is necessary to enhance surveillance not only for the early detection and implementation of response measures for WNV but also for other emerging flaviviruses that could be endemic in this area.

## Introduction

West Nile virus (WNV) is an important zoonotic virus with symptoms ranging from mild fever to severe lethal neuroinvasive disease in humans. The virus is maintained in an enzootic cycle among mosquitoes belonging to the *Culex* genus and birds, with mammals (equids and humans) being dead-end hosts. In humans, most of the infections are asymptomatic (80%) and those who develop disease, after an incubation period of 2 to 14 days, usually have mild symptoms such as fever, headache, fatigue, malaise, myalgia, arthralgia, rash, lymphadenopathy, and gastrointestinal symptoms (anorexia, nausea, vomiting, or diarrhoea) (Gray and Webb, 2014; Barzon et al., 2015; Bai et al., 2019). Less than 1% of infected cases develop a neuroinvasive disease (WNND) such as encephalitis, meningitis, and acute flaccid paralysis, frequently associated with risk factors such as aging, solid organ transplant, diabetes, hypertension, and other immunosuppression conditions related to fatal outcome (Lindsey et al., 2012). Moreover, atypical or rare presentations of WNV disease (WNVD) such us myocarditis, pancreatitis, hepatitis, cerebellitis, rhabdomyolysis, and ocular manifestations have also been described (Hasbun et al., 2016; Konjevoda et al., 2019; Velasco et al., 2020). WNVD has sometimes been associated with several sequelae, the most frequent being muscle weakness, fatigue, myalgia, memory loss, depression, and difficulty doing activities of daily living (Carson et al., 2006; Hughes et al., 2007). There is no vaccine for humans, and treatment is supportive as there are no specific antiviral drugs.

In humans, the peak of viraemia is 4–8 days post-infection (dpi). Antibodies can be detected in serum after 3–9 dpi, and the WNV IgM generally persists for over 6 months and may still be detectable for up to 1 year (Barzon et al., 2015). The laboratory-based diagnostic approaches are composed of virus isolation, RT-PCR, serology, and pathological examination. The most commonly used molecular diagnostic technique is real-time reverse transcription polymerase chain reaction (qRT-PCR), as it is a very fast and reliable technique, which also quantifies the viral genome. Serologically, diagnosis is based on the detection of IgM and IgG antibodies against WNV. As cross-reactivity with other flaviviruses may occur, the virus neutralisation tes by virus neutralisation tes against WNV remains the gold standard because of its high specificity and ability to detect and quantify neutralising antibodies to the virus (Tardei et al., 2000; Lustig et al., 2018). The limitation of this assay is that it takes a week to obtain results and, in Europe, a biosafety level 3 laboratory is required.

Since its discovery, WNV has caused human and animal disease outbreaks all over the world, except in Antarctica. In Europe, the virus is endemic and emerging in multiple countries. Over the last two decades, there have been notable increases in the number and extension of human and equine cases. Several genetic lineages of the virus have been detected (Vázquez et al., 2010), but lineages 1 and 2 have been mainly responsible for the disease in humans and equids in European countries. According to reports by the European Center for Disease Prevention and Control (ECDC), a significant increase in the number of human cases was observed in 2018 in Europe, eight times higher than in 2017 (ECDC, 2019). Most locally acquired cases were reported by Italy, Greece, and Romania, representing 39%, 20%, and 18% of EU cases, respectively. During 2020, EU/EEA and EU-neighbouring countries reported 336 locally acquired human cases of WNVD, mostly in Greece, Spain, and Italy (ECDC, 2020). Later, in 2021 (ECDC, 2022a) and 2022 (ECDC, 2022b), 139 and 965 human cases respectively were reported in Europe. In Spain, WNV fever is a notifiable disease. The first two notified human cases were detected in Andalusia in 2010 (García-Bocanegra et al., 2011), although the first known human case—diagnosed retrospectively—occurred in 2004 in Badajoz (Extremadura) (Kaptoul et al., 2007). Three additional cases were notified in Andalusia in 2016 (López-Ruiz et al., 2018), and no autochthonous human cases were reported from 2017 to 2019 in Spain. In the summer of 2020, the greatest number of WNV cases in humans in Spain was described (García San Miguel Rodríguez-Alarcón et al., 2021), with 77 cases detected in southwest Spain (71 from Andalusia and 6 from Extremadura) in areas where the virus was detected in previous years in humans, animals, and/or mosquitoes. Between 2021 and 2022, 10 human cases were reported in Spain, eight in Andalusia, and two in Catalonia, but none in Extremadura (ISCIII, 2022). Several studies in Spain have revealed the circulation of WNV in Andalusia during at least the last two decades. Moreover, the presence of WNV through seroprevalence studies is being detected in other Spanish regions (Extremadura, Catalonia, Castilla La Mancha, Castilla León, Comunidad Valenciana, and Mallorca) in birds, horses, and other mammals (Vanhomwegen et al., 2017; Napp et al., 2021; Casades-Martí et al., 2023).

In 2020 in Extremadura, where WNV human cases had not been reported since 2004, six autochthonous WNVD were described in Badajoz province. These cases were detected in areas where circulation of WNV and other flaviviruses, such as Usutu (USUV) and Tick-borne encephalitis (TBEV), has been described in birds, horses, and/or dogs (García-Bocanegra et al., 2018; Bravo-Barriga et al., 2021; Guerrero-Carvajal et al., 2021). Moreover, in 2020, when the WNV human outbreak occurred, USUV RNA was detected in mosquitoes and USUV-specific antibodies were detected in wild birds close to rural and urban areas, which is indicative of an active circulation and represents a public health threat (Bravo-Barriga et al., 2023). In 2021, no WNV human cases or animal outbreaks were reported through the veterinary surveillance system, and in 2022, there were only three equine outbreaks in Badajoz (Ministry of Agriculture and Fisheries and Food, 2023a; Ministry of Agriculture and Fisheries and Food, 2023b).

## Materials and methods

### Outbreak detection

On 22/09/2020, the National Reference Laboratory for Arboviruses in Spain (NRL), of the National Center for Microbiology (NCM-ISCIII), confirmed a WNV human case in a patient in the province of Badajoz (Extremadura). In the following 4 weeks, five more cases were detected in surrounding areas, one of them by additional epidemiological investigations and retrospective serological analyses from patients with neurological disease of unknown but suspected viral aetiology. No travel history was described for the six detected WNV human cases.

The data available from the Ministry of Agriculture and Fisheries and Food (Ministry of Agriculture and Fisheries and Food, 2023a; Ministry of Agriculture and Fisheries and Food, 2023b) regarding animal surveillance and human cases were consulted, and a map was made with spatial distribution in livestock regions (sanitary areas concerning veterinary health). This map was created with free software QGIS v.3.18.

### Case definition

In Spain, WNVD has been notifiable since 2010. The detection of a single case is considered a Public Health Alert. Epidemiological data are provided as soon as it is available and updated according to the evolution of the cases, following National Guidelines, which contain case definition, public health measures such us seasonal active surveillance of meningoencephalitis cases in certain regions considered at risk, and response in case of an outbreak together with standardised survey of cases. According to the National Guidelines and the European Union case definition, a WNVD human case is suspected when a person lives in/or has visited a high-risk area, or has been bitten by mosquitoes and presents at least one of the following signs or symptoms with or without fever (>38.5°C): encephalitis, meningitis, acute flaccid paralysis, or Guillain-Barré syndrome. Laboratory case definitions and diagnostic algorithms for WNV human infections are defined as laboratory-confirmed or probable cases. At least one laboratory criterion is required to confirm the case: isolation of the virus, nucleic acid detection in a clinical sample, IgM detection in cerebrospinal fluid (CSF), or WNV IgM and IgG detection in sera confirmed by the neutralisation assay. The presence of WNV-specific antibodies in a serum sample allows only probable case classification. Laboratory results need to be interpreted according to flavivirus vaccination status (European Commission, 2018; ISCIII, 2021).

### Ethical statement

The cases reported in this study were investigated with routine procedures according to the national surveillance plan for WNV infection. A unique ID to ensure the anonymity of patients and no patient identifiers were included in the study. The study was approved by the Ethical Committee of ISCIII (No. CEI PI 06_2023).

### Microbiological investigations

In the regional hospitals, the acute serum samples were tested by serological methods to detect recent infection due to *Borrelia* spp., *Leptospira* spp. and *Coxiella burnetii*, and by molecular methods to detect herpes simplex virus, varicella zoster virus, enterovirus, and cytomegalovirus in the central nervous system (CNS). The CSF samples were also biochemically analysed.

For WNV diagnosis, the samples were sent to the NCM-ISCIII. Molecular and serological methods were used in serum, urine, and CSF samples. The presence of WNV RNA was investigated in serum, urine, and CSF acute samples using a specific WNV qRT-PCR (Vázquez et al., 2016). Anti-WNV immunoglobulin M (IgM) and immunoglobulin G (IgG) antibodies were determined in human CSF by sera acute and convalescent samples, using WNV IgM Capture DxSelect and WNV IgG DxSelect ELISA kits (Focus Diagnostics, Cypress, California, USA).

Specimens found positive for WNV antibodies were also tested against other flaviviruses (USUV and TBEV) to exclude possible cross-reactivity. The methods used were USU IgG ELISA Euroimmun assay and TBE IgG and IgM indirect immunofluorescence assays (IFA) (Flavivirus Mosaic 1, Euroimmun, Lübeck, Germany) for TBEV. These assays were performed according to the manufacturer’s instructions. WNV IgM-positive results were confirmed performing the WNV IgM assay, which was carried out in parallel in the presence and absence of antigen.

To confirm the specificity of the antibody response, positive or indeterminate sera in both WNV IgG and IgM ELISA tests were assayed by neutralisation test (NT) against WN (strain HU6365/08), USU (strain HU10279/09), and TBE (strain Neudorfl) viruses. For this purpose, samples were tested in duplicate. Briefly, serum samples were inactivated at 56°C for 30 min and then twofold dilutions (25 µl) of the samples ranging from 1:8 to 1:512 were placed in a 96-well tissue culture microplate (Nunc A/S, Roskilde, Denmark) and mixed with 25 µl containing 100 tissue culture infectivity doses (100 TCID_50_) of the virus. After 1 h of incubation in a 5% CO_2_ incubator at 37°C, 50 µl of a Vero E6 cell suspension containing 4 × 10^5^ cells/ml was added to each well. Cultures were maintained for 7 days at 37°C and 5% CO_2_, and microscopic evaluation of the cytopathic effect was carried out 3, 5, and 7 days after inoculation. The titres of neutralising antibodies were defined as the highest serum dilution that showed >90% neutralisation of the virus challenge. Neutralising antibody titres ≥1:16 were considered positive. Specific responses to viruses were based on the comparison of NT titres obtained in parallel against the three flaviviruses, and the neutralising immune response observed was considered specific when NT titres for a given virus were >fourfold higher than the titre obtained for the other viruses. All these procedures were performed in a biosafety level 3 laboratory.

The WNV NAT (Nucleic Acid Testing) screening in blood donations in Extremadura was performed on individual samples using the commercial cobas^®^ WNV Test on the cobas 6800 System (Roche Diagnostics). This screening was performed until the end of the WNV season attending to the recommendations of official organisms.

## Results

### Description of human cases of WNV infection

Between September and October 2020, six human cases of WNV infection were identified. The first WNV case was diagnosed on 22 September and the last one on 16 October. Symptom onset occurred between 1 and 30 September. All the patients were over 50 years old, and the average age was 64 (range 51 to 80). Two were women and four were men. No epidemiological link according to municipality of infection was found. All cases were admitted to the hospital and presented fever and other WNVD-related symptoms such as headache (n = 3), malaise (n = 2), dizziness (n = 2), diarrhoea (n = 1), vomiting (n = 1), cough (n = 1), hypertension (n = 1), and arthromyalgia (n = 1). Five of them presented WNND and the other one fever with arthromyalgia, headache, lymphopenia, and thrombopenia. Several complications such as asthenia, hypertension, unstable gait, muscle pains, bradypsychia, fever, dizziness, tremors, arthralgia, hydrocephalus, and cognitive impairment were observed, and three patients with previous pathologies presented worse disease evolution. There were no deaths, and all cases were discharged by 23 October. The mean length of stay was 16 days (range 6 to 29) (**Table 1**).

**Table 1:** Summary of clinical data of the patients infected with West Nile virus in Extremadura, Spain, 2020.

Patient ID	Age ranges (years)	Gender	Symptom onset (2020)	Clinical symptoms	Comorbidity	Evolution with complications
**Case 1**	50-60	Female	01/09/2020	Fever, malaise, diarrhoea, dizziness, meningoencephalitis	Yes	Yes
**Case 2**	50-60	Female	10/09/2020	Fever, malaise, headache, vomiting, meningoencephalitis	None	No
**Case 3**	70-79	Male	21/09/2020	Fever and neurological involvement	Yes	Yes
**Case 4**	50-60	Male	06/09/2020	Fever, headache, hypertension, dizziness, meningitis	None	Yes
**Case 5**	70-80	Male	30/09/2020	Fever, cough, meningoencephalitis	Yes	Yes
**Case 6**	70-80	Male	23/09/2020	Fever, arthromyalgia, headache	None	No

None of the patients had a history of travel abroad during the incubation period, and none had been vaccinated against other flaviviruses such as TBEV, yellow fever (YFV), or Japanese encephalitis (JEV).

Patients’ epidemiological, clinical, and laboratory data are presented in **Tables 1**, **2**.

**Table 2:** Summary of the molecular and serological results obtained from the analysis carried out in the samples obtained from the patients.

	Case 1				Case 2					Case 3			Case 4				Case 5				Case 6			
Sample	CSF	Urine	Serum	Serum	CSF	Serum	Urine	Serum	Serum	CSF	Serum	Serum	CSF	Urine	Serum	Serum	CSF	Urine	Serum	Serum	CSF	Serum	Urine	Serum
**days post symptom onset**	6	35	35	64	6	13	13	60	>120	6	7	>180	12	23	23	>60	6	8	8	12	13	13	23	30dpi
**qRT-PCR**	NEG	NEG	NEG	ND	NEG	NEG	NEG	ND	ND	NEG	NEG	ND	NEG	NEG	NEG	ND	NEG	NEG	NEG	ND	ND	ND	NEG	ND
**WNV IgM**	**POS (5.5)**	ND	**POS (4.3)**	**POS (2.7)**	**POS (5)**	**POS (7.5)**	ND	**POS (2.02)**	**POS (1.15)**	**POS (3.3)**	**POS (4.7)**	**POS (3.44)**	**POS (3.6)**	ND	**POS (3.6)**	**POS (2.4)**	NEG (0.5)	ND	**POS (5.7)**	**POS (4.84)**	NEG (0.6)	**POS (5.6)**	ND	**POS (6.5)**
**WNV IgG**	NEG (1.1)	ND	**POS (4.8)**	**POS (4.7)**	**POS (1.9)**	**POS (2.8)**	ND	**POS (4.54)**	**POS (5.04)**	NEG (0.9)	**POS (1.9)**	**POS (4.43)**	NEG (1)	ND	**POS (2.74)**	**POS (4.1)**	NEG (0.1)	ND	**POS (2.2)**	**POS (4.34)**	NEG (0)	NEG (1)	ND	**POS (2.2)**
**WNV NT**	ND	ND	**1/64**	**1/64**	ND	**1/16**	ND	**1/64**	**1/32**	NEG_NEG	**1/16**	**1/64**		ND	**1/16**	**1/32**		ND	1/8	**1/64**	ND	1/8	ND	**1/16**
**USUV IgG**	NEG			**POS (1.3)**	NEG				**POS (1.1)**	NEG		**POS (1.2)**	NEG			**POS (1.3)**	NEG			**POS (1.5)**	NEG			NEG (0.5)
**USUV NT**	ND	ND	ND	NEG	ND	ND	ND	NEG	NEG		NEG	NEG		ND		NEG		ND		NEG	ND	ND	ND	NEG
**IgM TBEV**	NEG		NEG			NEG		NEG		NEG		NEG	NEG			NEG			**POS**			NEG		
**IgG TBEV**				**POS**					**POS**			**POS**				**POS**				**POS**				**POS**
**NT TBEV**				NEG					NEG			NEG				NEG				NEG				NEG

CSF, cerebrospinal fluid; ELISA, enzyme-linked immunosorbent assay; IFA, immunofluorescence assay; qRT-PCR, real-time RT-PCR; ND, test was not done; NEG, negative; NT, neutralising antibody titres; POS, positive; WNV, West Nile virus; USUV, Usutu virus; TBEV, tick-borne encephalitis virus

Bold values mean that the result is positive.

### Laboratory results

In total, 24 samples belonging to the six patients were analysed. The acute serum samples were negative for detecting recent infection due to *Borrelia* spp., *Leptospira* spp., *Coxiella burnetii*, herpes simplex virus, varicella zoster virus, enterovirus, and cytomegalovirus. In the CSF, the bacterial culture was negative and the CSF analysis typically showed mononuclear pleocytosis (>15 leucocytes/µl) with elevated protein concentration and a normal glucose level.

There were 15 samples (sera, CSF and urine) examined by qRT-PCR for WNV, none of which were positive (**Table 2**). All the cases were confirmed by detection of neutralising antibodies against WNV by the neutralisation assay in serum samples, although in four out of six cases, diagnosis was also confirmed by detection of IgM antibodies in CSF. The titre of the neutralising WNV antibodies was low, in a range from 1/16 to 1/64. A convalescent serum was obtained in four patients, and a slight rise in the neutralisation titre was found. All the human serum samples showed IgM positivity for WNV and seroconversion or a rise of the IgG level. IgM against TBEV was also tested in some acute and convalescent serum samples by IFI giving negative results, except one sample that showed positive immunofluorescent signal at 8 days post-symptom onset (dpo). However, all convalescent samples IgG positive for WNV showed cross-reactivity in IgG against TBEV and USUV, showing a high degree of cross-reactivity in these assays between these flaviviruses, but no neutralising antibodies against TBEV or USUV were detected.

### Outbreak control measures

After the first human case diagnosis on 22 September, a control of the blood and organs donation system was activated, according to the Ministry of Health regulations, the National Transplant Organization, and the European Commission directive 2014/110/UE (Scientific Committee for Transfusion Safety, 2021). Regarding the measures adopted to prevent the transmission of the virus through blood donations in risk areas, once the first confirmed WNV case was detected, all donations in the affected areas were blocked during the incubation period of the first case. Moreover, blood collections were cancelled in high-risk areas until September 29 when WNV NAT screening was introduced. This measure to control donations was maintained until the end of the virus circulation season. All the samples tested by NAT screening were negative. Moreover, an active surveillance was established by epidemiological surveillance units and Hospital Clinical Services, and all clinicians were alerted through the Extremadura Health Service. A retrospective search for possible WNVD human cases (undiagnosed meningitis and meningoencephalitis from Badajoz province) was intensified after the first case, identifying five probable cases of which one was confirmed by laboratory methods. Additional measures were activated, including information campaigns on mosquito bite prevention, cleaning potential mosquito-breeding sites, recommendations for tissue-sample handling, postmortem examination, and safety in transfusions and transplants. Additionally, at a Regional level, an entomological surveillance was established. Furthermore, at a National level, a review and update of the national guidelines for surveillance and rapid risk assessments was performed (Spanish Ministry of Health, 2020).

**Figure 1:**
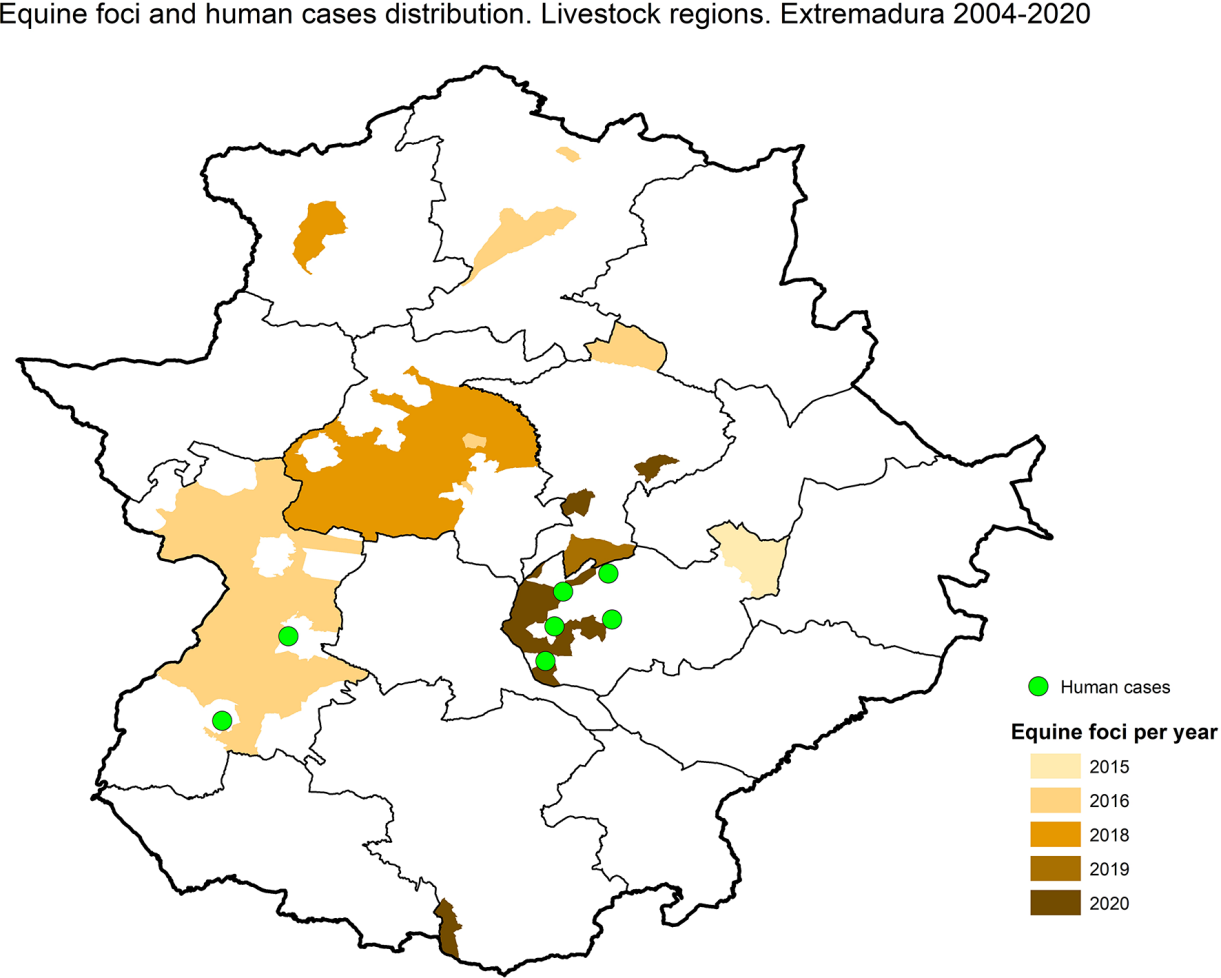
Areas with WNV equine foci or human cases. Data from 2004 to 2020.

According to the data available from the Ministry of Agriculture and Fisheries and Food (2023a) and (2023b) regarding animal surveillance, seven cases of WNV were reported in horses belonging to three livestock regions (sanitary areas concerning veterinary health), between 4 September and 23 October in six areas in Extremadura (Caceres and Badajoz), all very close to the affected area where the human cases were detected (**Figure 1**). Of the seven horses, five (71%) were detected in Badajoz province and two (29%) in Caceres province. Five of them (71%) were diagnosed in September and three of them a few days before the first human case (**Table 3**).

**Table 3:** Epidemiological information of the equine and human cases in Extremadura, Spain.

Number	Province	Livestock regions (National foci)	Sensitive animals	Affected animals	Confirmation date
Equine foci					
2022/3	Badajoz	Don Benito		3	
2020/136	Cáceres	Trujillo	27	1	23/10/2020
2020/107	Badajoz	Don Benito	24	1	05/10/2020
2020/103	Badajoz	Zafra	3	1	30/09/2020
2020/102	Badajoz	Zafra	2	1	25/09/2020
2020/88	Cáceres	Trujillo	14	1	16/09/2020
2020/50	Badajoz	Don Benito	15	1	04/09/2020
2020/51	Badajoz	Don Benito	1	1	04/09/2020
2019/6	Cáceres	Trujillo	3	1	28/11/2019
2018/6	Cáceres	Coria	17	1	23/10/2018
2018/4	Cáceres	Caceres	1	1	22/10/2018
2016/72	Badajoz	Badajoz	7	1	15/11/2016
2016/70	Badajoz	Badajoz	37	1	15/11/2016
2016/67	Cáceres	Plasencia	7	1	25/10/2016
2016/44	Cáceres	Plasencia	14	1	04/10/2016
2016/43	Cáceres	Caceres	4	1	04/10/2016
2016/42	Cáceres	Trujillo	10	1	23/09/2016
2015/4	Badajoz	Don Benito	1	1	25/09/2015
Human cases					
Case 1/2020	Badajoz	Don Benito			16/10/2020
Case 2/2020	Badajoz	Don Benito			22/09/2020
Case 3/2020	Badajoz	Badajoz			05/10/2020
Case 4/2020	Badajoz	Don Benito			15/10/2020
Case 5/2020	Badajoz	Don Benito			26/10/2020
case 6/2020	Badajoz	Don Benito			16/10/2020
First case	Badajoz	Badajoz			2004

## Discussion

During the 2020 WNV transmission season, Spain was the second country with more human WNV infections in Europe, registering the highest human WNV outbreak ever in the country with a total of 77 cases. Six patients were infected in Extremadura, the region where the first autochthonous human case was retrospectively identified in 2004 and no other cases had been reported until 2020. The absence of human cases reported in previous seasons in this area could be explained by a lack of clinical suspicion as most infections are asymptomatic (80%). Even when WNV infection is suspected, the diagnosis is complicated and a combination of molecular and serological assays is necessary. Generally, patients with severe clinical symptoms seek medical care, therefore the probability of detecting and reporting them is higher. In this outbreak, the six described cases exhibited fever, the most common clinical signs of the infection, and five of them (83%) presented neurological disease. Taking into account that less than 1% of infected cases develop a WNND, a high number of WNV infections would have gone undiagnosed. Thus, educational plans targeting citizens, clinicians, public health workers, and decision makers should be carried out in order to increase the awareness of WNV and other emerging diseases.

WNVD in humans is associated with mild symptoms (fever, headache, chill, malaise, myalgia, arthralgia, rash, vomiting, nausea, anorexia), most of which were present in the patients described in this outbreak. However, atypical or rare presentations such as myocarditis, pancreatitis, hepatitis, cerebellitis, rhabdomyolysis, and ocular manifestations were not observed even though the patients described in this work presented several of the risk factors related with worse disease evolution and aftermath such as the age (ranging between 50 and 80), living in rural areas in contact with animals, diabetes, hypertension, cardiovascular disease, and chronic lymphocytic leukaemia. In fact, several sequelae have been described in some of these patients such as unstable gait, muscle pains, bradipsychia, dizziness, tremors, forgetfulness, asthenia, hydrocephalus, arthralgia, muscle paints, dyspnoea, and cognitive impairment, especially in patients with chronic morbidities. In general, age and gender are the main intrinsic predisposing factors related to WNVD. It has been reported that individuals older than 50 years of age are more susceptible to severe infections with neurological involvement and that patients older than 75 generally succumb to the infection (Gray and Webb, 2014). The latter could be explained by age-related innate immunosenescence. Regarding gender, more men have been described among WNVD cases, probably because they have more outdoor occupations and are more exposed to mosquito bites. In fact, in rural areas, WNV outbreaks and disease incidence and prevalence have been linked with agricultural activities. In the outbreak described in this work, although there were more men infected, we cannot draw conclusions due to the small number of cases. Other major risk factors are preexisting medical conditions such as cancers, cardiovascular diseases, and diabetes (chronic morbidities), and immunosuppressed individuals have a 40 times higher risk of contracting the disease and dying from WNV infection (Lindsey et al., 2012).

WNV diagnosis was possible in all cases by serological assays. From all patients, CSF, sera, and urine samples were available and analysed. Although all acute samples (<7 dpo) were available in four of the six patients, no positive results were obtained by qRT-PCR. Urine samples from different dpo (from 8 to 35) were analysed by PCR because in several studies viral RNA was detected in urine for much longer (including up to a month postinfection) than in plasma and with higher viral load (Barzon et al., 2014). However, all of the urine samples analysed in this work were negative.

In Extremadura, viral information about the WNV human and equine cases has not been available yet, even though sporadic WNV outbreaks in horses have been reported at least since 2015 (Ministry of Agriculture and Fisheries and Food, 2023a; Ministry of Agriculture and Fisheries and Food, 2023b). A recent study carried out in birds of this region (Bravo-Barriga et al., 2021) revealed WNV lineage 1 circulation, the same lineage that was detected previously in neighbouring areas in birds (Castilla La Mancha, 2007), mosquitoes (Andalusia, 2008; and 2020), and horses (Andalusia, 2010) (Sotelo et al., 2009; García-Bocanegra et al., 2011; Vázquez et al., 2011). These studies showed a high prevalence (18.23%) and active circulation of WNV in wild birds during the period 2017–2019. These seroprevalence levels are higher than those found in other European countries and very similar to those detected in birds from Doñana National Park, an endemic WNV area in Andalusia (López et al., 2011). Another study carried out in dogs in the southwest of Spain revealed the presence of neutralising antibodies against WNV and TBEV, demonstrating the circulation of these viruses in these areas (García-Bocanegra et al., 2018). Moreover, the recent detection of USUV (RNA in mosquitoes and USUV-specific antibodies in birds) and an undetermined flavivirus highlights the widespread circulation of WNV in this endemic area and its co-circulation with USUV and other flaviviruses (Bravo-Barriga et al., 2023). In the six patients studied in this work, cross-reactive IgG antibodies against USUV and TBEV were detected in all the convalescent sera samples tested, but not in the CSF. Although the presence of cross-reactive antibodies against the three flaviviruses was observed in the ELISA and IFA assays, confirmation of WNV infection was possible by neutralisation assays. Taking into account flavivirus co-circulation and the serological cross-reactivity described among these viruses, the laboratory findings should be carefully addressed and a combination of molecular and serological techniques is necessary for a complete diagnosis of this infection. Therefore, although no human infections by USUV or TBEV have been reported in Extremadura until now, when a human WNV infection is suspected in this area, a differential diagnosis should be carried out against these flaviviruses—which may also be endemic in this area—and serological cross-reactions should be excluded.

The history of detection of WNV for more than 15 years both in birds and horses, as well as the increase in human cases described in this work, indicates its establishment and spread in Extremadura, Spain. Moreover, the recent detection of USUV close to urban areas represents a public health threat that requires inclusion in the differential diagnoses in patients with compatible symptoms. Therefore, it is necessary to establish surveillance programs for these emerging flaviviruses and to develop coordinated national plans integrating multisectoral and interregional participation from a one-health approach.

## Data availability statement

The original contributions presented in the study are included in the article/supplementary material. Further inquiries can be directed to the corresponding author.

## Ethics statement

The studies involving human participants were reviewed and approved by Instituto de Salud Carlos III. Written informed consent for participation was not required for this study in accordance with the national legislation and the institutional requirements.

## Author contributions

AV coordinated the study. AM, PM, and SR performed the laboratory screening and biochemical analysis in the regional Hospitals. AV, LH, MS-S, and MP-O performed the laboratory analysis, confirmation, and interpretation of results. AntV, MG, MJ and CM-P performed the clinical case management and description. BFM participated in the epidemiological investigations. DG-B designed and graphed the map of human and animal cases. JR, EF, ED, and AC contributed to the surveillance and epidemiological investigations. All authors contributed to the article and approved the submitted version.
